# Citrullinated histone H3, a marker of extracellular trap formation, is increased in blood of stable asthma patients

**DOI:** 10.1186/s13601-020-00337-8

**Published:** 2020-07-13

**Authors:** Pawel Kuczia, Joanna Zuk, Teresa Iwaniec, Jerzy Soja, Jerzy Dropinski, Marta Malesa-Wlodzik, Lech Zareba, Jan G. Bazan, Anetta Undas, Stanislawa Bazan-Socha

**Affiliations:** 1 Department of Internal Medicine, Jagiellonian University Medical College, 8 Skawinska Str, 31-066 Kraków, Poland; 2 Allergology and Pulmonology Clinic, Institute of Tuberculosis and Lung Diseases, Regional Branch in Rabka-Zdrój, Rabka-Zdrój, Poland; 3 College of Natural Sciences, Institute of Computer Science, University of Rzeszow, 1 Pigonia Str., 35-310 Rzeszow, Poland; 4 Institute of Cardiology, Jagiellonian University Medical College, Kraków, Poland

**Keywords:** Asthma, Citrullination, Histone H3, Extracellular traps

## Abstract

**Background:**

Emerging data indicates that extracellular traps (ETs), structures formed by various immune cell types, may contribute to the pathology of noninfectious inflammatory diseases. Histone hypercitrullination is an important step in ETs formation and citrullinated histone H3 (H3cit) is considered a novel and specific biomarker of that process. In the present study we have evaluated circulating H3cit in stable asthmatics and investigated its relationship with asthma severity, pulmonary function and selected blood and bronchoalveolar lavage (BAL) biomarkers.

**Methods:**

In 60 white adult stable asthmatics and 50 well-matched controls we measured serum levels of H3cit. In asthmatics we also performed bronchoscopy with BAL. We analyzed blood and BAL biomarkers, including interleukin (IL)-4, IL-5, IL-6, IL-10, IL-12p70, IL-17A and interferon γ. For statistical analysis, Mann–Whitney U-test, χ^2^ test, one-way ANCOVA, ROC curve analysis and univariate linear regression were applied. Independent determinants of H3cit were established in a multiple linear regression model.

**Results:**

Asthma was characterized by elevated circulating H3cit (17.49 [11.25–22.58] vs. 13.66 [8.66–18.87] ng/ml, p = 0.03). In asthmatics positive associations were demonstrated between serum H3cit and lung function variables, including total lung capacity (TLC) (β = 0.37 [95% CI 0.24–0.50]) and residual volume (β = 0.38 [95% CI 0.25–0.51]). H3cit was increased in asthma patients receiving systemic steroids (p = 0.02), as well as in subjects with BAL eosinophilia above 144 cells/ml (p = 0.02). In asthmatics, but not in controls, circulating H3cit correlated well with number of neutrophils (β = 0.31 [95% CI 0.19–0.44]) and monocytes (β = 0.42 [95% CI 0.29–0.55]) in peripheral blood. Furthermore, BAL macrophages, BAL neutrophils, TLC, high-sensitivity C-reactive protein, Il-12p70 and bronchial obstruction degree were independent determinants of H3cit in a multivariate linear regression model.

**Conclusions:**

Asthma is characterized by increased circulating H3cit likely related to the enhanced lung ETs formation. Inhibition of ETs might be a therapeutic option in selected asthma phenotypes, such as neutrophilic asthma.

## Introduction

Asthma is a common chronic inflammatory disease of the airways with a complex pathomechanism involving diverse immune processes [1, 2] accompanied by a low-grade persistent systemic inflammation [3]. Emerging data indicate that extracellular traps (ETs) may contribute to the pathology of noninfectious inflammatory diseases [4–6]. ETs are web-like structures coated with histones, granular and cytosolic proteins, formed by various immune cells, including neutrophils, eosinophils, and macrophages [7]. Neutrophil ETs (NETs) were first discovered as being mainly implicated in the innate immune response in host defense, enabling neutrophils to immobilize and kill invading bacteria, fungi or even viruses [6, 8]. The impact and presence of ETs formation in asthma have not been extensively studied yet, however, previous studies suggest that NETs [9, 10] and eosinophil extracellular traps (EETs) [4, 11] may contribute to the persistent airway inflammation in asthma. Moreover, Toussaint et al. [12] have shown that rhinovirus respiratory tract infection, the most common cause of asthma exacerbation in humans, induces host-derived double strand DNA release in nasal lavage samples of patients with mild-moderate asthma.

There is also a growing body of evidence that ETs may be entangled in clotting of the blood. We have recently reported evidence of a prothrombotic state in asthma which is characterized by enhanced plasma thrombin formation, impaired clot lysis and platelet activation [13], all of them related to the low-grade systemic inflammation [3], endothelial injury [14], elevated exacerbation rate [15], and likely increased atherosclerotic risk [16, 17]. Higher risk of thromboembolic events in asthmatics has been also demonstrated in epidemiological studies [18–22]. The mechanisms functionally underlying this phenomenon are currently under investigation; however, ETs contribution may be of importance.

One of the key steps in ETs formation is citrullination of histones performed by the histone-specific enzyme peptidylarginine deiminase 4 (PAD4) [8]. Emerging data indicates that citrullinated histone H3 (H3cit), a key component of ETs, may be recognized as a specific marker of ETs formation both in tissue samples [23–25] and in peripheral blood [26–29]. Taking into account low-grade systemic inflammation demonstrated in asthma and important role of ETs formation in human pathology, we sought to evaluate serum H3cit, a specific biomarker of ETs formation, in asthma. We also studied its relation to asthma severity, lung function abnormalities, and selected blood and bronchoalveolar lavage asthma biomarkers.

## Methods

### Patients

The study was conducted in the Department of Internal Medicine, Jagiellonian University Medical College, Krakow, Poland, from June 2015 to January 2018. We enrolled 60 white adult patients with clinically stable asthma according to the Global Initiative for Asthma (GINA) guidelines [30] and 50 well-matched controls. The study participants were 18–65 years old. Diagnosis of asthma was established based on a history of recurrent respiratory symptoms (wheeze, cough, shortness of breath, and chest tightness) together with current and/or historically documented postbronchodilator increase in forced expiratory volume in one second (FEV_1_) of 12% and at least 200 ml from the baseline [30]. Atopic status was confirmed by a positive skin prick testing for at least one inhaled allergen (Allergopharma, Reinbeck, Germany). All asthma medications, except for biological therapy, were permitted, including oral corticosteroids at a daily dose equivalent to ≤ 10 mg of prednisolone, only if the dose was unchanged for 3 consecutive months. Asthma patients were eligible if they had no exacerbation during the preceding 6 months. Severity of asthma was categorized according to the GINA guidelines [30]. “Mild” asthma was defined as a mild persistent disease, treated with short acting β_2_-agonist on demand, with or without low daily dose of inhaled corticosteroids (ICS) (< 250 μg of fluticasone propionate [FP] [dry powder inhaler] or equivalent). “Moderate” asthma was defined as a mild persistent disease treated with a low (combined with long-acting β_2_-agonists) or medium dose of ICS (250–500 μg of FP or equivalent). “Severe” asthma was defined as severe persistent disease despite using high daily dose of ICS (> 500 μg of FP or equivalent). Asthma symptom control was assessed based on result of Asthma Control Test (ACT). Scores 20–25 were classified as “well-controlled asthma”, 16–19 as “not well-controlled”, while 5–15 as “very poorly controlled asthma”. Spirometry and bronchial reversibility test with 400 μg of albuterol as well as postbronchodilator body plethysmography with assessment of residual volume (RV) and total lung capacity (TLC) were measured in all enrolled asthma patients according to the American Thoracic Society (ATS) standards [31], using a Jaeger MasterLab spirometer (Jaeger-Toennies GmbH; Hochberg, Germany). In asthma patients we also performed bronchoscopy with bronchoalveolar lavage (BAL).

Exclusion criteria comprised any acute illness, congestive heart failure, atrial fibrillation, coronary heart disease, hyper- or hypothyroidism, liver injury, chronic kidney disease (stage 3 or more), autoimmune disease, malignant neoplasm or medical history of thromboembolism. In turn, arterial hypertension, diabetes mellitus, or hypercholesterolemia were allowed as comorbidities in subjects studied. Arterial hypertension was defined based on a history of hypertension (blood pressure > 140/90 mmHg), or present antihypertensive treatment. Diabetes mellitus was defined as the current use of insulin, or oral hypoglycemic medications, or fasting serum glucose > 7.0 mmol/l. Hypercholesterolemia was defined as serum total cholesterol > 5.2 mmol/l or previous diagnosis along with lipid-lowering treatment. Ex-smokers were eligible for enrolment if they had stopped smoking at least 5 years before inclusion.

### Controls

Control subjects were enrolled from the hospital personnel and its relatives. They were matched with patients according to age, sex, BMI, smoking status and internal medicine comorbidities. Subjects with history of allergic diseases or bronchial obstruction were excluded. Each control was individually matched with two patients considering the closest values of the matching criteria.

### Bronchofiberoscopy

Bronchofiberoscopy was performed in asthma subjects according to the ATS guidelines [32] using the BF 1T180 bronchoscope (Olympus, USA) with local anesthesia (2% lidocaine) and mild sedation (0.05–0.1 mg fentanyl and 2.5–5 mg midazolam, both intravenously). BAL was performed with 200 ml of normal saline applied into the right middle lobe bronchus. First 10 ml aliquot of obtained BAL fluid was discarded, while the next sample was used for investigation.

### BAL cellularity analysis

The cytospin preparations (Thermo Scientific, Walthman, MA) were made from BAL fluid samples and stained with the May-Grunwald Giemsa dye. Percentages of inflammatory cells among 1000 cells in each preparation were counted (with exception of epithelial cells). Remaining BAL fluid was centrifuged 2000×*g* for 20 min at room temperature, supernatant was frozen in aliquots and stored at − 70 °C until analysis.

### Laboratory investigations

Fasting blood samples were drawn from the antecubital vein between 8:00 and 11:00 A.M, using minimal stasis. Lipid profile, glucose, creatinine, urea, alanine aminotransferase, as well as complete blood cell and platelet count were assayed by routine laboratory techniques. Fibrinogen was determined with the Clauss method. High-sensitivity C-reactive protein (hsCRP) and immunoglobulin E (IgE) were measured by latex nephelometry (Siemens, Marburg, Germany). Blood samples were drawn into serum separation tube, centrifuged 2000×*g* for 20 min, at room temperature. The supernatant was frozen in aliquots and stored at − 70 °C until analysis.

High sensitivity immunoenzymatic assays were used to measure levels of interleukin(IL)-4, IL-5, IL-6, IL-10, IL-12p70, IL-17A, and interferon (INF)γ (eBiosciencea, Vienna, Austria, all) in serum and BAL fluid of asthmatics and in serum of 25 (50%) controls.

Concentration of H3cit in serum was measured using ELISA kit developed by Cayman Chemicals (Ann Arbor, MI, USA). This assay employed a monoclonal antibody specific for histone H3 citrullinated at R2, R8, and R17 (clone 11D3). The lower limit detection of the assay was 0.1 ng/ml, the upper 31 ng/ml.

## Statistical analysis

Analyses were carried out using Statistica software package version 12.5 (TIBCO Inc). The Shapiro–Wilk test has shown that continuous variables were non-normally distributed. They were reported here as median and interquartile range and compared using the Mann–Whitney U-test. Categorical variables were given as percentages and compared by χ^2^ test with Yates’ correction, if applicable. Age, sex, and body mass index (BMI) were considered as potential confounders for laboratory investigations. Therefore, the Box-Cox normality transformation was used and a one way covariance analysis (ANCOVA) was performed to adjust for confounding factors. To test for associations between two continuous variables univariate linear regression model was applied with adjustment for sex, age, and BMI. Independent determinants of H3cit were established in a multiple linear regression model, built by a forward stepwise selection procedure, verified by F Snedecor’s statistics, with F > 1. The R2 was used as a measure of the variance. Cut-off points of BAL and blood biomarkers in relation to circulating H3cit levels were calculated in asthmatics based on receiver operating characteristic (ROC) curves. Moreover, to compare biomarkers between H3cit-high and H3cit-low asthma subjects the 75^th^ percentile value of the circulating H3cit in asthma individuals has been taken into account. In each case of multiple comparisons Bonferroni correction has been applied and the nominal level of significance has been reduced proportional to the total number of all tests performed in multiple comparisons procedure. Results were considered significant when the p value was less than 0.05.

## Results

### Patients and controls

Clinical and laboratory characteristics of subjects studied are given in Tables 1 and 2, respectively.

**Table 1:** Clinical characteristics of subjects studied

	Asthma n = 60	Controls n = 50	p-value
Age, years	50 (39–56)	45.5 (39–53)	NS
Female gender	44 (73.7)	29 (58.0)	NS
Height, m	1.63 (1.58–1.71)	1.65 (1.6–1.76)	NS
Body mass index (BMI), kg/m^2^	26.9 (23.4–31.6)	26.4 (23.0–28.1)	NS
History of smoking	18 (30.0)	17 (34.0)	NS
Active smokers	7 (11.7)	8 (16.0)	NS
Arterial hypertension	27 (45.0)	16 (32.0)	NS
Diabetes mellitus	8 (13.3)	5 (10.0)	NS
Hypercholesterolemia	17 (28.3)	16 (32.0)	NS

Medications used:			
Inhaled corticosteroids	59 (98.3)		
Long-acting β2-agonists	49 (81.7)		
Montelukast	9 (15.0)		
Oral corticosteroids	17 (28.3)		
Atopy	41 (68.3)		
Asthma duration, years	10 (5–15)		

Asthma control (according to Asthma Control Test results)			
Well-controlled (score > 19)	15 (25.0)		
very poorly controlled (score > 16)	25 (41.7)		

Asthma severity (GINA):			
Mild	9 (15.0)		
Moderate	21 (35.0)		
Severe	30 (50.0)		
Prebronchodilator FEV_1_/VC, %	67.8 (59.4–75.9)		
Postbronchodilator FEV_1_/VC, %	73.1 (68.2–79.7)		
Postbronchodilator TLC, L [% of predicted value]	5.76 (5.18–6.47) [111.8 (103.3–119.8)]		
Postbronchodilator RV, L [% of predicted value]	2.23 (1.67–2.66) [123.6 (108.3–147.2)]		

Categorical variables are presented as numbers (percentages), continuous variables as median (interquartile range)

**Table 2:** Peripheral blood basic laboratory results

	Asthma n = 60	Controls n = 50	p-svalue
White blood cells, 10^3^/μl	6.82 (5.69–8.18)	5.73 (5.09–6.96)	0.003
Neutrophils, 10^3^/μl	3.39 (2.80–4.35)	3.3 (3.0–3.45)	NS
Eosinophils, 1/μl	285 (140–470)	150 (90–240)	< 0.001
Monocytes, 10^3^/μl	0.62 (0.50–0.88)	0.43 (0.40–0.49)	< 0.001
Lymphocytes, 10^3^/μl	2.04 (1.66–2.50)	1.69 (1.44–2.16)	0.02
Blood platelets, 10^3^/μl	230 (194–265)	224 (203–269)	NS
Hemoglobin, g/dl	13.0 (13.0–14.5)	13.8 (12.8–15.0)	NS
C-reactive protein, mg/l	1.64 (0.51–6.63)	1.02 (0.91–3.33)	NS
IgE, IU/ml	152.0 (34.1–532.0)	30.0 (17.8–37.0)	0.01
Alanine aminotransferase, IU/l	28 (22–42)	22 (14–28)	< 0.001
Total cholesterol, mmol/l	4.9 (4.1–5.6)	4.7 (4.1–5.2)	NS
Low density lipoprotein cholesterol, mmol/l	2.6 (1.9–3.1)	2.98 (2.48–3.51)	0.02
High density lipoprotein cholesterol, mmol/l	1.3 (1.1–1.6)	1.51 (1.25–1.84)	NS
Triglycerides, mmol/l	1.4 (1.2–2.0)	0.9 (0.7–1.4)	0.003

Variables are presented as median and interquartile range

**Fig. 1:**
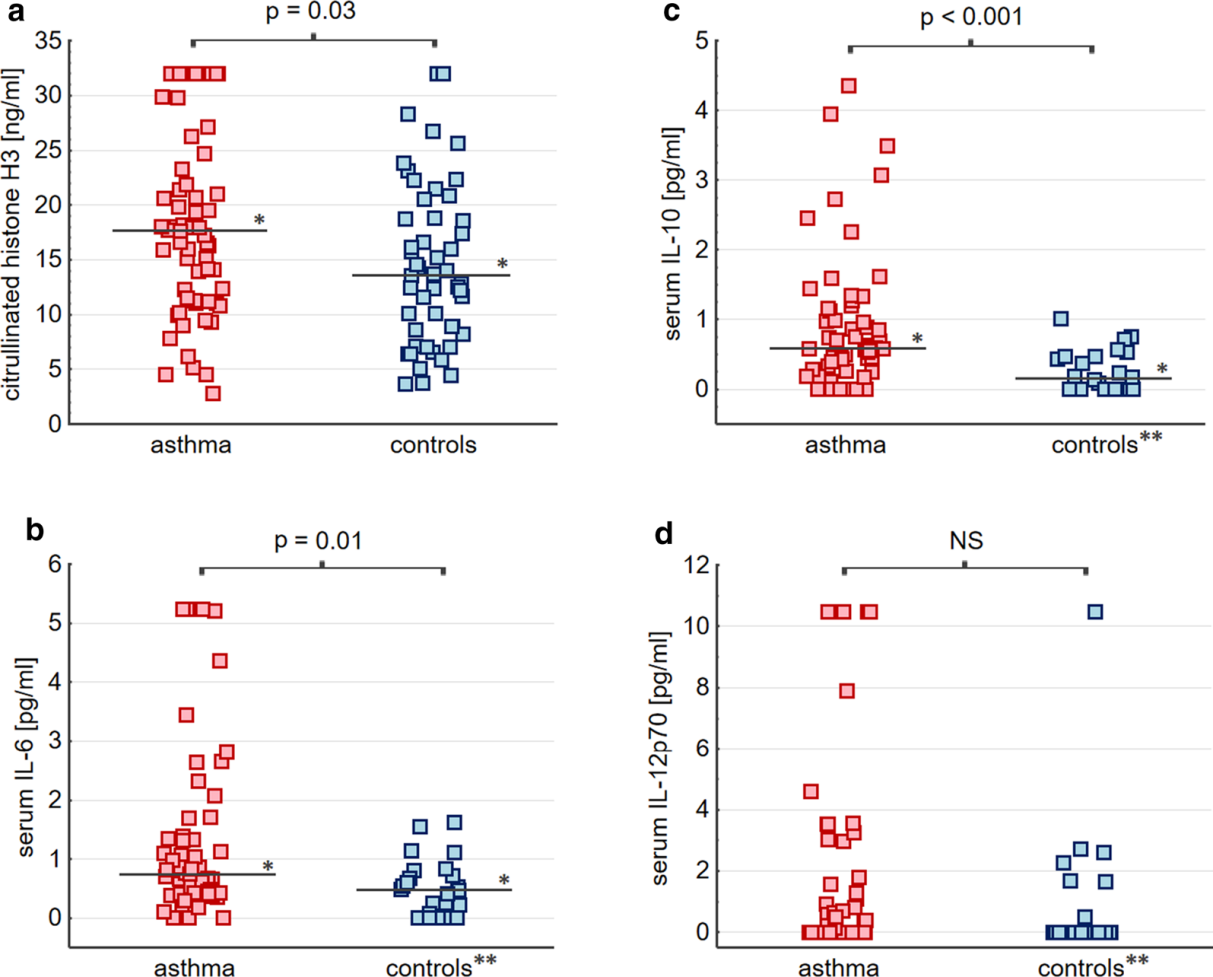
Circulating H3cit and relevant serum cytokine concentrations in asthma and control subjects. * Median value. ** Results available in 25 (50%) of control subjects. Serum H3cit was above the limit of detection (LoD) in all subjects studied (**a**). Serum IL-6 was above the LoD in 56 (93.3%) asthmatics and 18 controls (**b**). Serum IL-10 was above the LoD in 51 (85.0%) asthmatics and 14 controls (**c**). Serum IL-12p70 was above the LoD in 27 (45.0%) asthmatics and 7 controls (**d**)

Among asthmatics 41 (68.3%) subjects had atopy, while 30 (50%) were characterized by severe disease according to GINA [30]. Severe asthma was an indication for the use of systemic corticosteroids in 17 patients (28.3%). Asthma individuals were characterized by increased serum levels of IL-6, IL-10 (Fig. 1b, c), alanine aminotransferase activity, as well as elevated monocyte, eosinophil, lymphocyte, and total white blood cell (WBC) counts as compared to controls (Table 1). Results of laboratory BAL investigations are presented in Table 3. As it has been demonstrated, IL-6 and IL-12p70 were above the detection threshold in the majority of asthma patients. In turn, concentrations of IL-4, IL-5, IL-17A, and IFNγ in BAL and blood, as well as IL-10 in BAL were below the detection threshold in the majority of subjects (Additional file 1: Tables S1 and S2).

**Table 3:** Results of BAL investigations in asthmatics

BAL cellularity	
Macrophages, number of cells per 1 ml	8763 (5697–16,119)
Lymphocytes, number of cells per 1 ml	943 (561–1969)
Neutrophils, number of cells per 1 ml	297 (187–497)
Eosinophils, number of cells per 1 ml	80 (8–196)

BAL fluid cytokines	
IL-6, pg/ml	0.736 (0.153–1.116), above the LoD in 47 (78.3%) subjects
IL-12 (p70), pg/ml	0.081 (0.052–0.118), above the LoD in 58 (96.7%) subjects

Variables are presented as median and interquartile range

As expected, in asthma concentration of IL-6 in BAL was associated with BAL and blood neutrophilia (β = 0.51 [95% CI 0.41–0.6] and β = 0.41 [95% CI 0.31–0.51], respectively).

### Citrullinated histone H3 in peripheral blood

Circulating H3cit was significantly elevated in asthmatics in comparison to control group (17.49 [11.25–22.58] vs. 13.66 [8.66–18.87] ng/ml, p = 0.03), also after adjustment for potential confounders (p = 0.02) (Fig. 1a). Interestingly, women with asthma had 17% lower H3cit than male patients (16.21 [10.96–20.86] vs. 19.48 [15.86–30.97] ng/ml, p = 0.03). Also only in asthma we documented a negative association between H3cit and BMI (β = − 0.28 [95% CI − 0.4 to − 0.15]).

Serum H3cit was not related to the ACT score and did not differ among asthma severity subtypes, albeit it was significantly elevated in patients receiving systemic steroids (21.07 [15.9–32.0] vs 16.21 [10.52–20.27] ng/ml, p = 0.02) and in those on antileukotriene medications (24.76 [17.28–29.83] vs 16.36 [11.11–19.90] ng/ml, p = 0.04). Administration of other drugs, as well as comorbidities had no impact on that parameter.

### Laboratory variables and circulating H3cit

**Fig. 2:**
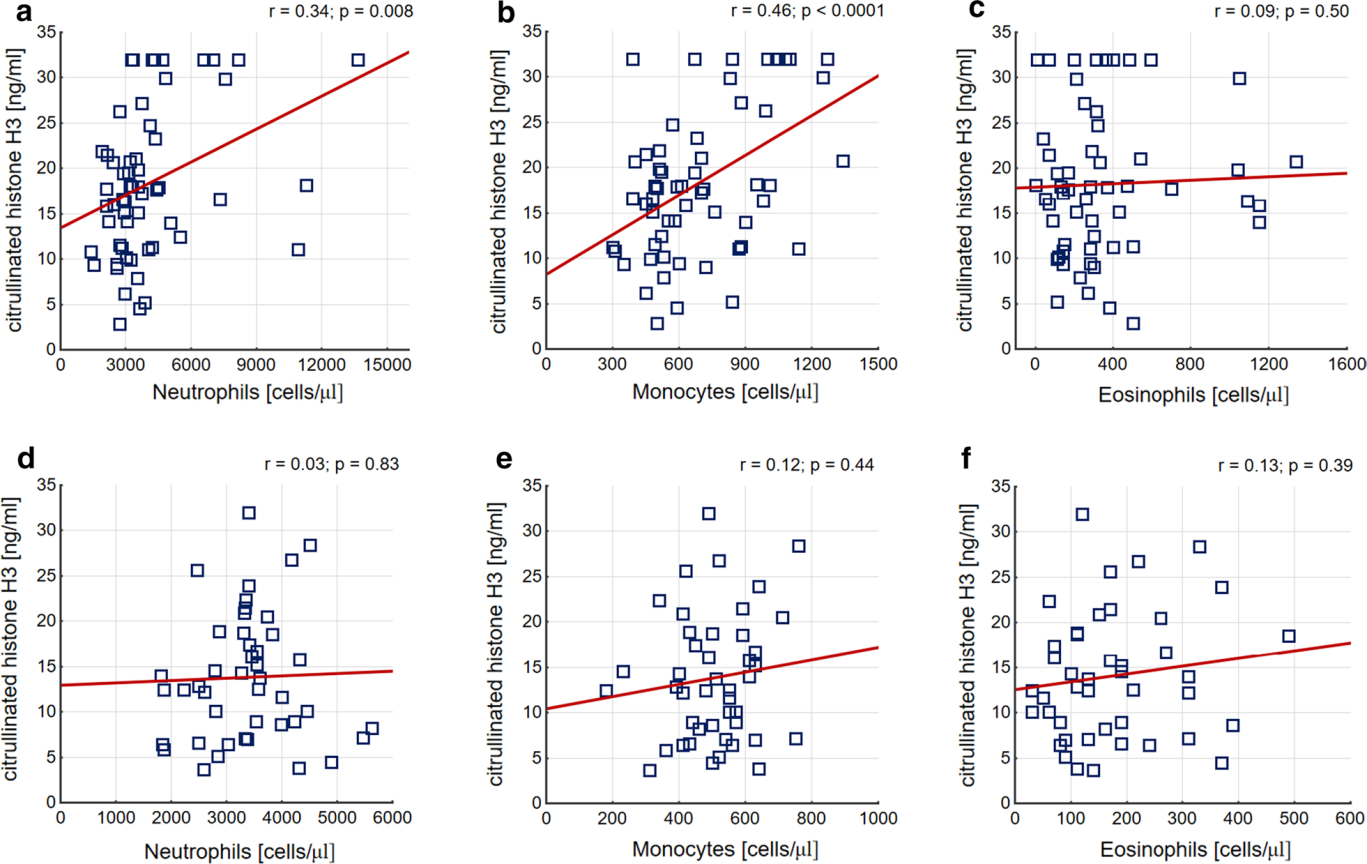
Correlations between peripheral blood cell counts and serum H3cit concentration. **a**–**c** Present correlations in asthma patients. **d**–**f** Present correlations in controls. Pearsons’s correlation coefficients with p-values were calculated and are shown in the panels

In asthma, but not in controls, circulating H3cit remained in positive associations with number of monocytes (β = 0.42 [95% CI 0.29–0.55]) and neutrophils (β = 0.31 [95% CI 0.19–0.44]) in peripheral blood (Fig. 2). Interestingly, in the patient group it also correlated with number of macrophages in BAL (β = 0.27 [95% CI 0.14–0.4]). Asthma patients with higher H3cit, defined as a value above 22.58 ng/ml (75th percentile in asthmatics) were characterized by increased monocyte (880 [680–1080] vs. 550 [480–720] cells/μl; p = 0.0009) and neutrophil (4350 [3730–7000] vs. 3120 [2580–3740] cells/μl; p = 0.0005) cell count in peripheral blood.

Moreover, using ROC curve analysis to calculate cut-off points we found that serum H3cit was increased in subjects with BAL eosinophilia above 144 cells/ml (20.77 [18.26–23.27] ng/ml vs. 16.05 [14.57–17.54] ng/ml, p = 0.02), with BAL total cell count higher than 10.725 cells/ml (18.09 [11.29–29.83] ng/ml vs. 15.12 [9.48–19.53] ng/ml, p = 0.03), as well as with BAL IL-6 levels higher than 0.76 pg/ml (19.53 [11.29–32.00] ng/ml vs. 15.98 [10.96–18.06] ng/ml, p = 0.04) (Additional file 2: Fig. S1). Interestingly, in controls H3cit was directly associated with serum IL-6 (β = 0.33 [95% CI 0.1–0.56]).

### Lung function tests and circulating H3cit

In asthmatics, serum H3cit was strongly related to the results of lung function tests, including TLC (β = 0.37 [95% CI 0.24–0.50]) and RV (β = 0.38 [95% CI 0.25 to 0.51]), also after adjustment for potential confounders. Asthma patients with higher H3cit, defined as values above 22.58 ng/ml (75th percentile as the cut-off point) had higher TLC in comparison to the remainders (6.42 [5.82–7.33] vs. 5.55 [5.11–6.14]; p = 0.007) (Additional file 3: Fig. S2c). Moreover, a weak negative relationship was demonstrated between H3cit and prebronchodilator FEV_1_/vital capacity (VC) index (β = −0.19 [95% CI − 0.32 to − 0.07]).

### Multiple regression model to predict circulating H3cit

Independent determinants of circulating H3cit in asthmatics were identified using forward stepwise selection procedure. The number of macrophages and neutrophils in BAL, hsCRP, TLC, and the degree of prebronchodilator airway obstruction were demonstrated as positive predictors of H3cit level in peripheral blood. In turn, surprisingly, IL-12 (p70) had a negative impact on circulating H3cit in that analysis. All aforementioned variables explained 57% of H3cit variability (Table 4).

**Table 4:** Independent determinants of circulating H3cit in asthmatics

	β	95% CI
FEV1/VC, %	− 0.15	− 0.28 to − 0.03
TLC, L	0.41	0.28 to 0.54
Macrophages, number per 1 ml of BAL fluid	0.21	0.07 to 0.34
Neutrophils, number per 1 ml of BAL fluid	0.36	0.24 to 0.50
IL-12(p70) in serum, pg/ml	− 0.18	− 0.32 to − 0.05
hsCRP, mg/l	0.37	0.22 to 0.51
F = 6.65, R^2^ = 0.58, p = 0.0002		

The resulting standardized regression coefficient (β) with 95% confidence intervals (95% CI) for a factor (independent variable) indicates the increase/decrease in standard deviations (SDs) of dependent variable, when that particular factor increases with 1 SD and all other variables in the model are unchanged

## Discussion

In the present study we have demonstrated that serum H3cit, a novel biomarker of ETs formation, is increased in stable asthma subjects. Although serum H3cit was not related to the asthma severity according to the GINA guidelines, a weak negative correlation with FEV_1_/VC suggests that enhanced bronchial obstruction, characteristic for more severe asthma subtype, might be associated with ETs release. The same applies to the tendency towards elevated circulating H3cit in those on systemic steroids or even anti-leukotriene medications. To our knowledge there is no evidence for upregulation of ETs formation by steroids [33] or leukotriene antagonists, however we cannot exclude such a relationship.

Previously, Dworski et al. [11] have found evidence for lung EETs and NETs formation in endobronchial biopsy specimens of mild and well-controlled asthma patients. In our study, the positive correlations demonstrated for TLC or RV and circulating H3cit suggest that indeed, lungs might be the site of ongoing ETs formation with subsequent H3cit release into the peripheral blood of stable asthmatics. It might be assumed that in asthma higher lung volume and capacity, thus larger inflammatory site area, might result in the increase of local ETs generation.

Interestingly, in asthma serum H3cit was related to the number of monocytes and neutrophils in peripheral blood, as well as macrophages in BAL. It is an intriguing result, as the issue of extracellular trap formation by macrophages remains a subject of ongoing research and debate [34]. On the other hand, we found no direct association between serum H3cit and number of neutrophils in BAL. That finding might be to some extent explained by the relatively low percentage of patients with neutrophilic BAL in our cohort, as other studies demonstrated increased NETs in neutrophilic asthma sputum [10]. However, we found increased H3cit among asthmatics with higher BAL eosinophilia. Previously, Choi et al. [4] have demonstrated likely impact of eosinophils’ derived EETs on airway inflammation. Our results stay in line with their finding, although BAL composition does not necessarily reflect lung tissue inflammation [11].

Associations between circulating H3cit and blood monocytes or neutrophils were not directly documented in the multivariate linear regression model, as hsCRP, a biomarker closely related to monocyte and neutrophil counts in our study (data not shown), was a more precise determinant of serum H3cit. Moreover, in that model BAL macrophages and BAL neutrophils remained independent H3cit determinants, suggesting the role of H3cit as a potential biomarker of neutrophilic asthma even despite the lack of a direct correlation between BAL neutrophils and H3cit.

Interestingly, in our asthma cohort higher concentrations of BAL IL-6 was associated with increased circulating H3cit. In turn, elevated serum IL-6 in asthmatics stays in line with the previous reports of persistent low-grade systemic inflammation in asthma. Links between NETs and cytokine release have already been documented, e.g. NETs may induce the transcription of IL-6 and pro-IL-1β genes in macrophages in early atherosclerosis [8]. Surprisingly, in our study asthma patients were characterized by IL-10 elevation as compared to controls. IL-10 is rather known for its anti-inflammatory properties including inhibition of NETs release [8]. Moreover, Borish et al. have found diminished IL-10 BAL fluid concentrations in asthmatics [35]. However, the observed increase in serum IL-10 in our study together with the negative correlation between BMI and H3cit in asthma subjects make further research of interactions between cytokines, adiponectin and ETs release of interest.

Interestingly, serum H3cit was negatively determined by serum IL-12(p70), a major player in the initiation and maintenance of T helper 1 cells response [36]. IL-12 is known to inhibit airway inflammation and bronchial obstruction in murine models of allergic asthma [37]. An interventional study with recombinant IL-12 in human asthmatics showed a decrease in peripheral blood and sputum eosinophilia with no impact on bronchial reactivity [38]. Another function of IL-12 is the inhibition of pathological angiogenesis [39]. It has been demonstrated that abnormal neovascularization is an important feature of airway remodeling in asthma patients [40, 41]. We cannot exclude, that in asthmatics with enhanced ETs formation decrease of IL-12 might contribute to the unfavorable airway remodeling and bronchial neovascularization.

Notably, increased H3cit in peripheral blood of asthma subjects is particularly important, since there is a growing body of evidence that enhanced ETs formation might contribute to the development of various systemic sequelae, including thrombotic complications or autoimmunity [6] [51]. In particular, the presence of autoantibodies against citrullinated peptides in rheumatoid arthritis, another noninfectious inflammatory disease, is likely related to the PAD4 activity enabling ETs release [6, 42]. In turn, severe asthma patients present more frequently with autoimmune diseases [52]. Thus, production of ETs with subsequent release of related products into the circulation may provide the initial stimulus for autoimmunity, explaining the increased rate of autoimmune diseases in asthmatics.

Moreover, it has been demonstrated that histones might directly contribute to inflammatory injury by a host tissue damage [43–46], including microvascular endothelial barrier disruption and subsequent cell inflow into the inflammatory site [43, 47, 48]. Experimental approach with specific H3cit inhibition led to improved outcomes in mouse sepsis models [29, 49]. Although this is only a hypothesis, we believe that even relatively low concentrations of H3cit and other citrullinated histone types may contribute to the endothelium damage in asthmatics. The availability of pharmacologic histone inhibitors makes it a particularly intriguing issue, as their use might be of benefit at least in some asthma phenotypes.

Furthermore, the potential contribution of ETs formation to the prothrombotic phenomenon in asthma also deserves a comment. Fuchs et al. [50] has reported that NETs trigger the blood clotting through activation of platelets, recruitment of red blood cells and stimulation of fibrin deposition, being a potential link between immune response and thrombosis. In particular, Ammollo et al. [51] revealed that extracellular histones increase plasma thrombin generation by impairing thrombomodulin-dependent protein C activation. Recently, Zuo et al. [52] documented increased H3cit and myeloperoxidase-DNA (MPO-DNA) in sera of subjects with coronavirus disease 2019 (COVID-19), illness accompanied by clotting impairment and microvascular thrombosis. Thus, we speculate that elevated circulating H3cit may, at least to some extent, explain prothrombotic blood alterations and increased risk of thromboembolic complications demonstrated even in well-controlled asthma patients [13, 18–20].

The limitations of our study include relatively small sample size. Particularly, subgroup analysis should be interpreted with caution. We determined each variable at a single time point, thus we cannot exclude changes of the variables during time. We did not analyze ETs formation in a functional assay. However, the laboratory techniques for directly determining the ETs formation are difficult and very demanding from the technical point of view. Applied here H3cit ELISA assay is simple, but relatively novel, thus might require further validation before being established as a reliable test for the extensive use in practice. We analyzed H3cit in blood and not in airways, which are the main loci of asthma inflammatory response. We did not measure other cytokines potentially relevant for the lung and systemic inflammatory response, including IL-1β and IL-18, released after NLRP3 stimulation by NETs [53]. Statistical associations reported here might not necessarily indicate cause-effect relationships. Finally, a clinical relevance of increased serum H3cit in relation to asthma course, disease progression, exacerbation risk, or vascular outcomes remains to be established.

## Conclusion

Asthma is characterized by increased circulating H3cit likely related to the enhanced lung ETs formation. Although large experimental and observational studies are needed to verify this hypothesis, it seems that ETs release is involved in asthma pathogenesis. Thus inhibition of ETs might be a therapeutic option in selected asthma phenotypes, such as neutrophilic asthma.

## Supplementary information

**Additional file 1. Table S1.** Cytokine concentrations in peripheral blood of asthma and control subjects (all tested cytokines). **Table S2.** BAL cytokine concentrations in asthmatics (all tested cytokines).

**Additional file 2. Figure S1.** Comparisons of asthma patient subgroups distinguished on the basis of ROC curve analysis.

**Additional file 3. Figure S2.** Comparisons of H3cit-low vs. H3cit-high asthma patients. Asthma patients were subdivided into H3cit-high and H3cit-low groups using blood H3cit value of the 75th percentile as the cut-off point (22.58 ng/ml).

## Data Availability

The datasets used and/or analysed during the current study are available from the corresponding author on reasonable request.

## Abbreviations

ACT: Asthma Control Test
ANCOVA: Analysis of covariance
ATS: American Thoracic Society
BAL: Bronchoalveolar lavage
BMI: Body mass index
EETs: Eosinophil extracellular traps
ETs: Extracellular traps
FEV1: Forced expiratory volume in one second
FP: Fluticasone propionate
GINA: Global Initiative for Asthma
hsCRP: High-sensitivity C-reactive protein
H3cit: Citrullinated histone H3
IgE: Immunoglobulin E
LoD: Limit of detection
ICS: Inhaled corticosteroids
IL: Interleukin
INFγ: Interferon γ
NETs: Neutrophil extracellular traps
PAD4: Peptidylarginine deiminase 4
RV: Residual volume
TLC: Total lung capacity
WBC: White blood cell
VC: Vital capacity

## References

[1] Lambrecht BN, Hammad H (2015). The immunology of asthma. Nat Immunol.

[2] Kozlik P, Zuk J, Bartyzel S, Zarychta J, Okon K, Zareba L (2020). The relationship of airway structural changes to blood and bronchoalveolar lavage biomarkers, and lung function abnormalities in asthma. Clin Exp Allergy.

[3] Bazan-Socha S, Mastalerz L, Cybulska A, Zareba L, Kremers R, Zabczyk M (2017). Prothrombotic state in asthma is related to increased levels of inflammatory cytokines, IL-6 and TNFα. Peripher Blood Inflamm..

[4] Choi Y, Le Pham D, Lee DH, Lee SH, Kim SH, Park HS (2018). Biological function of eosinophil extracellular traps in patients with severe eosinophilic asthma. Exp Mol Med.

[5] Uddin M, Watz H, Malmgren A, Pedersen F (2019). NETopathic inflammation in chronic obstructive pulmonary disease and severe asthma. Front Immunol..

[6] Jorch SK, Kubes P (2017). An emerging role for neutrophil extracellular traps in noninfectious disease. Nat Med.

[7] Boeltz S, Amini P, Anders H-J, Andrade F, Bilyy R, Chatfield S (2019). To NET or not to NET:current opinions and state of the science regarding the formation of neutrophil extracellular traps. Cell Death Differ.

[8] Papayannopoulos V (2018). Neutrophil extracellular traps in immunity and disease. Nat Rev Immunol.

[9] Pham DL, Ban G-Y, Kim S-H, Shin YS, Ye Y-M, Chwae Y-J (2017). Neutrophil autophagy and extracellular DNA traps contribute to airway inflammation in severe asthma. Clin Exp Allergy.

[10] Wright TK, Gibson PG, Simpson JL, McDonald VM, Wood LG, Baines KJ (2016). Neutrophil extracellular traps are associated with inflammation in chronic airway disease. Respirology.

[11] Dworski R, Simon H-U, Hoskins A, Yousefi S (2011). Eosinophil and neutrophil extracellular DNA traps in human allergic asthmatic airways. J Allergy Clin Immunol..

[12] Toussaint M, Jackson DJ, Swieboda D, Guedán A, Tsourouktsoglou T-D, Ching YM (2017). Host DNA released by NETosis promotes rhinovirus-induced type-2 allergic asthma exacerbation. Nat Med.

[13] Bazan-Socha S, Mastalerz L, Cybulska A, Zareba L, Kremers R, Zabczyk M (2016). Asthma is associated with enhanced thrombin formation and impaired fibrinolysis. Clin Exp Allergy.

[14] Pacholczak R, Kuszmiersz P, Iwaniec T, Zaręba L, Zarychta J, Walocha J (2020). Endothelial dysfunction and pentraxin-3 in clinically stable adult asthma patients. J Investig Allergol Clin Immunol.

[15] Bazan-Socha S, Mastalerz L, Cybulska A, Zareba L, Kremers R, Zabczyk M (2017). Impaired fibrinolysis and lower levels of plasma α2-macroglobulin are associated with an increased risk of severe asthma exacerbations. Sci Rep..

[16] Bazan-Socha S, Kuczia P, Potaczek DP, Mastalerz L, Cybulska A, Zareba L (2018). Increased blood levels of cellular fibronectin in asthma: relation to the asthma severity, inflammation, and prothrombotic blood alterations. Respir Med.

[17] Kuczia P, Mastalerz L, Potaczek DP, Cybulska A, Zareba L, Bazan-Socha S (2019). Increased activity of lipoprotein-associated phospholipase A2 in non-severe asthma. Allergol Int..

[18] Majoor CJ, Kamphuisen PW, Zwinderman AH, Brinke A, Amelink M, Rijssenbeek-Nouwens L (2013). Risk of deep vein thrombosis and pulmonary embolism in asthma. Eur Respir J.

[19] Chung W-S, Lin C-L, Ho F-M, Li R-Y, Sung F-C, Kao C-H (2014). Asthma increases pulmonary thromboembolism risk: a nationwide population cohort study. Eur Respir J.

[20] Zöller B, Pirouzifard M, Memon AA, Sundquist J, Sundquist K (2017). Risk of pulmonary embolism and deep venous thrombosis in patients with asthma: a nationwide case–control study from Sweden. Eur Respir J.

[21] Onufrak SJ, Abramson JL, Austin HD, Holguin F, McClellan WM, Vaccarino LV (2008). Relation of adult-onset asthma to coronary heart disease and stroke. Am J Cardiol.

[22] Tattersall MC, Guo M, Korcarz CE, Gepner AD, Kaufman JD, Liu KJ (2015). Asthma predicts cardiovascular disease events. Arterioscler Thromb Vasc Biol.

[23] Masuda S, Nakazawa D, Shida H, Miyoshi A, Kusunoki Y, Tomaru U (2016). NETosis markers: quest for specific, objective, and quantitative markers. Clin Chim Acta.

[24] Muniz VS, Silva JC, Braga YAV, Melo RCN, Ueki S, Takeda M (2018). Eosinophils release extracellular DNA traps in response to Aspergillus fumigatus. J Allergy Clin Immunol..

[25] Pertiwi KR, de Boer OJ, Mackaaij C, Pabittei DR, de Winter RJ, Li X (2019). Extracellular traps derived from macrophages, mast cells, eosinophils and neutrophils are generated in a time-dependent manner during atherothrombosis. J Pathol..

[26] Thålin C, Daleskog M, Göransson SP, Schatzberg D, Lasselin J, Laska AC (2017). Validation of an enzyme-linked immunosorbent assay for the quantification of citrullinated histone H3 as a marker for neutrophil extracellular traps in human plasma. Immunol Res.

[27] Bryk AH, Prior SM, Plens K, Konieczynska M, Hohendorff J, Malecki M (2019). Predictors of neutrophil extracellular traps markers in type 2 diabetes mellitus: associations with a prothrombotic state and hypofibrinolysis. Cardiovasc Diabetol..

[28] Pan B, Alam HB, Chong W, Mobley J, Liu B, Deng Q (2017). CitH3: a reliable blood biomarker for diagnosis and treatment of endotoxic shock. Sci Rep..

[29] Biron BM, Chung C-S, O’Brien XM, Chen Y, Reichner JS, Ayala A (2017). Cl-Amidine prevents histone 3 citrullination and neutrophil extracellular trap formation, and improves survival in a murine sepsis model. J Innate Immun..

[30] Global Initiative for Asthma. Global Strategy for Asthma Management and Prevention. 2016. http://ginasthma.org/.

[31] Culver BH, Graham BL, Coates AL, Wanger J, Berry CE, Clarke PK (2017). Recommendations for a standardized pulmonary function report An official American Thoracic Society technical statement. Am J Respir Crit Care Med.

[32] Sokolowski JW, Burgher LW, Jones FL, Patterson JR, Selecky PA (1987). Position paper on guidelines for fiberoptic bronchoscopy in adults. Am Rev Respir Dis.

[33] Vargas A, Boivin R, Cano P, Murcia Y, Bazin I, Lavoie JP (2017). Neutrophil extracellular traps are downregulated by glucocorticosteroids in lungs in an equine model of asthma. Respir Res.

[34] Doster RS, Rogers LM, Gaddy JA, Aronoff DM (2018). Macrophage extracellular traps: a scoping review. J Innate Immun..

[35] Borish L, Aarons A, Rumbyrt J, Cvietusa P, Negri J, Wenzel S (1996). Interleukin-10 regulation in normal subjects and patients with asthma. J Allergy Clin Immunol..

[36] Jalah R, Rosati M, Ganneru B, Pilkington GR, Valentin A, Kulkarni V (2013). The p40 subunit of interleukin (IL)-12 promotes stabilization and export of the p35 subunit: implications for improved IL-12 cytokine production. J Biol Chem.

[37] Gavett SH, O’Hearn DJ, Li X, Huang SK, Finkelman FD, Wills-Karp M (1995). Interleukin 12 inhibits antigen-induced airway hyperresponsiveness, inflammation, and Th2 cytokine expression in mice. J Exp Med.

[38] Bryan SA, O’connor BJ, Matti S, Leckie MJ, Kanabar V, Khan J (2000). Effects of recombinant human interleukin-12 on eosinophils, airway hyper-responsiveness, and the late asthmatic response. Lancet.

[39] Tugues S, Burkhard SH, Ohs I, Vrohlings M, Nussbaum K, Vom Berg J (2015). New insights into IL-12-mediated tumor suppression. Cell Death Differ.

[40] Vrugt B, Wilson S, Bron A, Holgate ST, Djukanovic R, Aalbers R (2000). Bronchial angiogenesis in severe glucocorticoid-dependent asthma. Eur Respir J.

[41] Wilson JW, Robertson CF (2002). Angiogenesis in paediatric airway disease. Paediatr Respir Rev.

[42] Koushik S, Joshi N, Nagaraju S, Mahmood S, Mudeenahally K, Padmavathy R (2017). PAD4: pathophysiology, current therapeutics and future perspective in rheumatoid arthritis. Expert Opin Ther Targets..

[43] Silk E, Zhao H, Weng H, Ma D (2017). The role of extracellular histone in organ injury. Cell Death Dis..

[44] Xu J, Zhang X, Pelayo R, Monestier M, Ammollo CT, Semeraro F (2009). Extracellular histones are major mediators of death in sepsis. Nat Med.

[45] Ekaney ML, Otto GP, Sossdorf M, Sponholz C, Boehringer M, Loesche W (2014). Impact of plasma histones in human sepsis and their contribution to cellular injury and inflammation. Crit Care.

[46] Alhamdi Y, Abrams ST, Cheng Z, Jing S, Su D, Liu Z (2015). Circulating histones are major mediators of cardiac injury in patients with sepsis. Crit Care Med.

[47] Meegan JE, Yang X, Beard RS, Jannaway M, Chatterjee V, Taylor-Clark TE (2018). Citrullinated histone 3 causes endothelial barrier dysfunction. Biochem Biophys Res Commun.

[48] Opal SM, van der Poll T (2015). Endothelial barrier dysfunction in septic shock. J Intern Med.

[49] Li Y, Liu Z, Liu B, Zhao T, Wang Y, Velmahos G (2014). Citrullinated histone H3—a novel target for treatment of septic shock. J Surg Res.

[50] Fuchs TA, Brill A, Duerschmied D, Schatzberg D, Monestier M, Myers DD (2010). Extracellular DNA traps promote thrombosis. Proc Natl Acad Sci.

[51] Ammollo CT, Semeraro F, Xu J, Esmon NL, Esmon CT (2011). Extracellular histones increase plasma thrombin generation by impairing thrombomodulin-dependent protein C activation. J Thromb Haemost.

[52] Zuo Y, Yalavarthi S, Shi H, Gockman K, Zuo M, Madison JA (2020). Neutrophil extracellular traps in COVID-19. JCI Insight..

[53] Tapia VS, Daniels MJD, Palazón-Riquelme P, Dewhurst M, Luheshi NM, Rivers-Auty J (2019). The three cytokines IL-1β, IL-18, and IL-1α share related but distinct secretory routes. J Biol Chem.

