# Supplementary material for: Citrullinated histone H3, a marker of extracellular trap formation, is increased in blood of stable asthma patients
Source: Clin Transl Allergy. 2020 Jul 13;10:31. doi: 10.1186/s13601-020-00337-8 (PMC7354860; doi:10.1186/s13601-020-00337-8)
Supplement: Supplementary file 1 — Additional file 1. Table S1. Cytokine concentrations in peripheral blood of asthma and control subjects (all tested cytokines). Table S2. BAL cytokine concentrations in asthmatics (all tested cytokines). [file 13601_2020_337_MOESM1_ESM.docx]

**Suppl. Table 1. Cytokine concentrations in peripheral blood of asthma and control subjects (all tested cytokines).**

|  | Asthma | Controls | p - value |
| --- | --- | --- | --- |
| IL-4, pg/ml | 0.005 (0.005-0.005)  above the LoD in 2 (3.3%) subjects | 0.005 (0.005-0.005)*  above the LoD in 0 subjects | NS |
| IL-5, pg/ml | 0.005 (0.005-0.005)  above the LoD in 0 subjects | 0.005 (0.005-0.005)*  above the LoD in 8 (32%) subjects | NS |
| IL-6, pg/ml | 0.750 (0.426-1.392)  above the LoD in 56 (93.3%) subjects | 0.487 (0.089-0.818)*  above the LoD in 18 (72%) subjects | 0.01 |
| IL-10, pg/ml | 0.585 (0.260-1.163)  above the LoD in 51 (85%) subjects | 0.174 (0.005-0.470)*  above the LoD in 14 (56%) subjects | <0.001 |
| IL-12 (p70), pg/ml | 0.005 (0.005-1.570)  above the LoD in 27 (45%) subjects | 0.005 (0.005-1.653)*  above the LoD in 7 (28%) subjects | NS |
| IL-17A, pg/ml | 0.005 (0.005-0.167)  above the LoD in 24 (40%) subjects | 0.005 (0.005-0.056)*  above the LoD in 6 (24%) subjects | NS |
| INF-γ, pg/ml | 0.005 (0.005-0.309)  above the LoD in 27 (45%) subjects | 0.005 (0.005-0.128)*  above the LoD in 11 (44%) subjects | NS |

Variables are presented as median and interquartile range.

LoD – limit of detection

*results available in 25 (50%) control subjects

**Suppl. Table 2. BAL cytokine concentrations in asthmatics (all tested cytokines).**

| BAL fluid cytokines | |
| --- | --- |
| IL-4, pg/ml | 0.005 (0.005-0.222), above the LoD in 19 (31.7%) subjects |
| IL-5, pg/ml | 0.005 (0.005-0.005), above the LoD in 1 (1.7%) subjects |
| IL-6, pg/ml | 0.736 (0.153-1.116), above the LoD in 47 (78.3%) subjects |
| IL-10, pg/ml | 0.005 (0.005-0.005), above the LoD in 5 (8.3%) subjects |
| IL-12 (p70), pg/ml | 0.081 (0.052-0.118), above the LoD in 58 (96.7%) subjects |
| IL-17A, pg/ml | 0.005 (0.005-0.005), above the LoD in 4 (6.7%) subjects |
| INF-γ, pg/ml | 0.005 (0.005-0.005), above the LoD in 1 (1.7%) subjects |

Variables are presented as median and interquartile range.

LoD – limit of detection
