# Supplementary figures and images for: Citrullinated histone H3, a marker of extracellular trap formation, is increased in blood of stable asthma patients
Source: Clin Transl Allergy. 2020 Jul 13;10:31. doi: 10.1186/s13601-020-00337-8 (PMC7354860; doi:10.1186/s13601-020-00337-8)

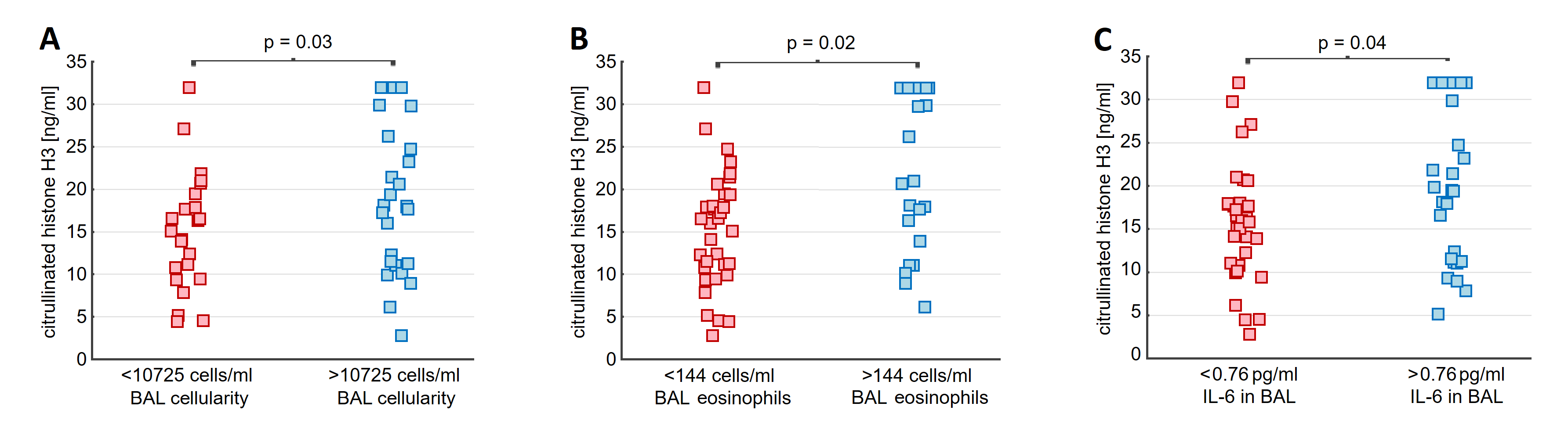

Supplement: Supplementary file 2 — Additional file 2. Figure S1. Comparisons of asthma patient subgroups distinguished on the basis of ROC curve analysis. [file 13601_2020_337_MOESM2_ESM.png]

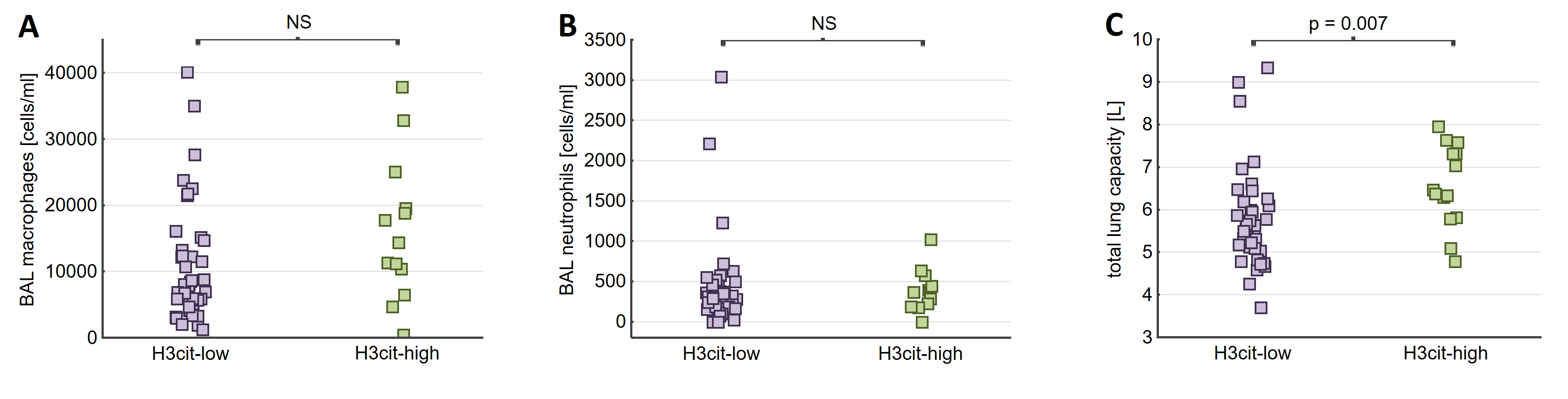

Supplement: Supplementary file 3 — Additional file 3. Figure S2. Comparisons of H3cit-low vs. H3cit-high asthma patients. Asthma patients were subdivided into H3cit-high and H3cit-low groups using blood H3cit value of the 75th percentile as the cut-off point (22.58 ng/ml). [file 13601_2020_337_MOESM3_ESM.png]
